# Physical Constraint Finite Element Model for Medical Image Registration

**DOI:** 10.1371/journal.pone.0140567

**Published:** 2015-10-23

**Authors:** Jingya Zhang, Jiajun Wang, Xiuying Wang, Xin Gao, Dagan Feng

**Affiliations:** 1 School of Electronic and Information Engineering, Soochow University, Suzhou 215006, P.R.China; 2 Changshu Inst Technol, Dept Phys, Changshu 215500, P.R.China; 3 Institute of Biomedical Engineering and Technology and School of Information Technologies, University of Sydney, Sydney, NSW 2006, Australia; 4 Suzhou Institute of Biomedical Engineering and Technology, Chinese Academy of Science, Suzhou 215006, P.R.China; 5 Med-X Research Institute, Shanghai Jiao Tong University, P.R.China; University of Minnesota, UNITED STATES

## Abstract

Due to being derived from linear assumption, most elastic body based non-rigid image registration algorithms are facing challenges for soft tissues with complex nonlinear behavior and with large deformations. To take into account the geometric nonlinearity of soft tissues, we propose a registration algorithm on the basis of Newtonian differential equation. The material behavior of soft tissues is modeled as St. Venant-Kirchhoff elasticity, and the nonlinearity of the continuum represents the quadratic term of the deformation gradient under the Green- St.Venant strain. In our algorithm, the elastic force is formulated as the derivative of the deformation energy with respect to the nodal displacement vectors of the finite element; the external force is determined by the registration similarity gradient flow which drives the floating image deforming to the equilibrium condition. We compared our approach to three other models: 1) the conventional linear elastic finite element model (FEM); 2) the dynamic elastic FEM; 3) the robust block matching (RBM) method. The registration accuracy was measured using three similarities: MSD (Mean Square Difference), NC (Normalized Correlation) and NMI (Normalized Mutual Information), and was also measured using the mean and max distance between the ground seeds and corresponding ones after registration. We validated our method on 60 image pairs including 30 medical image pairs with artificial deformation and 30 clinical image pairs for both the chest chemotherapy treatment in different periods and brain MRI normalization. Our method achieved a distance error of 0.320±0.138 mm in x direction and 0.326±0.111 mm in y direction, MSD of 41.96±13.74, NC of 0.9958±0.0019, NMI of 1.2962±0.0114 for images with large artificial deformations; and average NC of 0.9622±0.008 and NMI of 1.2764±0.0089 for the real clinical cases. Student’s t-test demonstrated that our model statistically outperformed the other methods in comparison (*p*-values <0.05).

## Introduction

Widely available biomedical images are essential for more accurate diagnosis and management of patients with a variety of diseases. Registration of these medical images into a spatial correspondence and alignment is important for fusion and maximization of the underlying information in the imaging datasets. Image registration is achieved by estimating an optimal transformation or displacement field [1]. It is one of the most fundamental research areas for various clinical applications, including planning and delivery of radiotherapy treatment [2–3], image-guided surgery [4–5], cross modality image fusion [6], morphometric study [7] and treatment monitoring [8].

After more than three decades of intensive research, various registration methods have been proposed [9–11]. Among these methods, the intensity based registration has the advantage of direct usage of the image intensity information without necessary preprocessing of segmentation and extraction of features such as salient points, curves and surfaces as in the feature based registration. However, compared with feature based counterpart, these intensity based methods normally face the challenges of reducing high computational cost and avoiding local optimization.

Basically, two types of geometric transformations are used in the intensity based methods which are derived from interpolation theory or derived from physical models. Free-form deformation (FFD) method [12] and local affine transformation (LAT) method [13] are two most common types of interpolation strategies. In FFD method, a rectangular grid of control points is defined in order to determine the deformation. Displacements between control points are propagated by interpolation. This method requires few degrees of freedom to describe local deformations and is able to efficiently provide smooth results. However, the dependence on a regular grid of control points restricts their adaptability and it is difficult to change control points topology. Many extensions of FFD methods have been proposed [14–15]. LAT methods parameterize the transformation by locally linear deformation. The images are hierarchically partitioned into contiguous blocks and an affine transformation is recovered for each one of them. However, the underlying image content has not been considered in the splitting process, and the regularization providing for the global smoothness in LAT method may lead to the ambiguous matching [16].

The diffusion model and the elastic body model in the framework of finite element method are the currently recognized physical models for medical image registration. The diffusion model upon the optical flow (OF) constraint describes deformations by assuming a constant brightness constraint of floating voxels [17–18]. However, it lacked a sound theoretical justification [9]. In finite element elastic model, the deformation of the image was modeled as an elastic body that is described by the Navier-Cauchy partial differential equation [19]. The gradient of the similarity measure is used as an external force field which tries to deform the floating image to fit the reference image configuration, while the internal elastic forces of the solid oppose the deformation. Thus, the floating image is deformed until the internal and external forces reach an equilibrium state. On the basis of a linear assumption [20], the governing equation can be simplified and the nodal displacement matrix can be obtained under the nodal external force matrix in the framework of finite element analysis method. However, the linear assumption may be not valid for soft tissues with complex nonlinear behavior and cannot accurately simulate large organ deformations [21].

To address the challenging issue in the elastic registration, in this paper, we propose a new registration method based on the St. Venant–Kirchhoff (VK) model that is the simplest hyper-elastic material model for representing the material behavior of soft tissues. Furthermore, we extend this model in a dynamic fashion by using the Newtonian differential equation [22–23]. The geometrical nonlinearity of the continuum is taken into account by considering the quadratic term of the deformation gradient in the strain tensor when deriving the elastic force. For a more efficient implementation of our proposed realistic model, we adopt a hierarchical (also referred to as a “global-to-local”) strategy in our registration algorithm.

## Methods and Materials

In the proposed deformable registration algorithm, the displacement field is obtained subject to external constraints imposed by the similarity metric of the image pair for registration. The equilibrium of our model is achieved by the minimization of the global energy functional that consists of elastic deformation energy and the energy enforced by the external image force.

### 2.1 Governing Equation for the Nonlinear Elastic Model

In the framework of the finite element analysis method, the displacement of any image pixel can be interpolated with the nodal displacement and the shape function. On the basis of an isotropic linear assumption, a matrix equation describing the motion of the elastic model can be expressed as:

$$K\cdot u=\hat{R}$$ (1)

where the stiffness matrix **K** is obtained from the mesh and elastic parameters. $\hat{R}$ is the nodal external force matrix obtained by optimizing the spatially encoded mutual information (SEMI), and **u** is the displacement vector of element nodes.

The linear elastic finite element model (LFEM) has an important limitation when coping with large deformations. To account for large deformations, we propose a nonlinear elastic model governed by the second order differential equation as below:

$$M\cdot \ddot{u}+C\cdot \dot{u}+\hat{F}=\hat{R}$$ (2)

where **M** and **C** are the matrices of the mass and damping, respectively. These two matrices are interrelated to each other with **C** = *α* **M** where *α* is a scale factor. The mass matrix **M** is chosen to be a diagonal matrix with diagonal elements as $M_{ii}^{el}=\frac{1}{3}\rho V^{el}$, where *V* ^*el*^ is the volume of element *el*, and *ρ* is the mass density.

The elastic force matrix $\hat{F}$ constrains the solution space and together with the external force, this elastic force guides optimization process to converge. Under the linear elastic condition, $\hat{F}=\mathrm{KU}$ according to Eq (1), thus Eq (2) is deduced to the linear elastic dynamic finite element model (DFEM) as below:

$$M\cdot \ddot{u}+C\cdot \dot{u}+K\cdot u=\hat{R}$$ (3)

**Fig 1:**
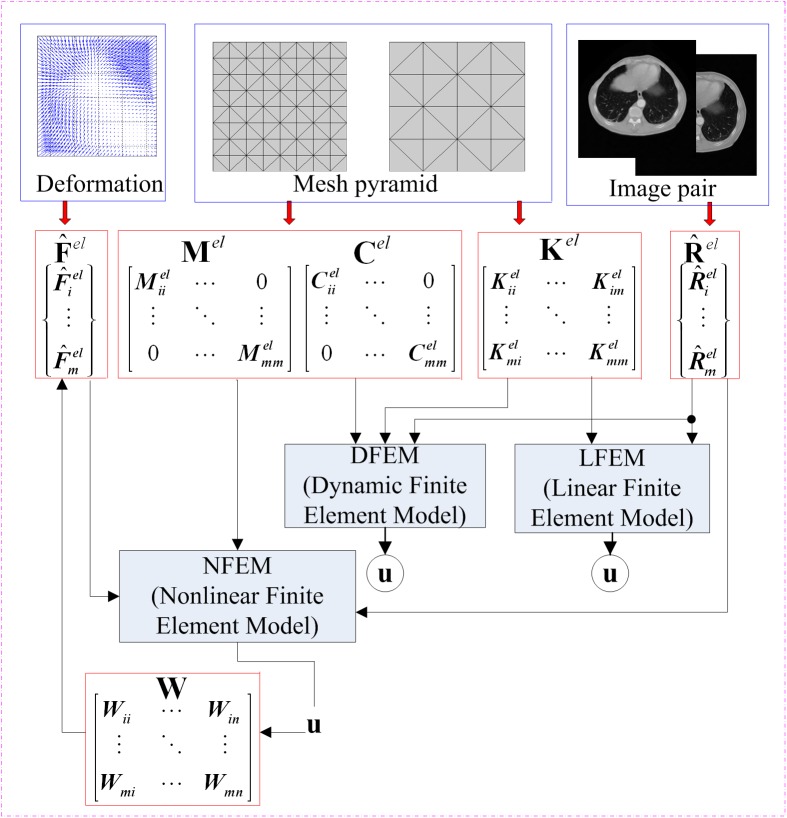
The component diagram of governing equations for LFEM, DFEM and NFEM.

In our proposed nonlinear finite element model (NFEM), the elastic force matrix $\hat{F}$ is derived from the elastic deformation energy and innovatively models the geometrical nonlinearity based on hyper-elastic material properties. Fig 1 illustrates the components governing equations for LFEM, DFEM and NFEM, and elaborates the differences of these three FEM registration methods.

The nonlinear governing Eq (2) is solved with an explicit integration scheme [24], where the displacement vector at time *t+*1 is computed from the elastic force and external force estimated at time *t* as follows:

$$(\frac{M}{\Delta t^{2}}+\frac{C}{2\Delta t})u^{t+1}=\hat{R}-\hat{F}+\frac{2M}{\Delta t^{2}}u^{t}+(\frac{C}{2\Delta t}-\frac{M}{\Delta t^{2}})u^{t-1}$$ (4)

where △*t* is the integration time step.

### 2.2 Modeling of Elasticity via Green-St.Venant Strain Measure

The hyperelastic material model has been proven appropriate to represent the material behavior of soft tissues and has been successfully applied in surgery simulation [25–26]. We used the simplest isotropic hyperelastic material model as proposed by Wittek et al. [27]. In this paper, we derive the elastic force $\hat{F}$ to model tissue deformation on the basis of St.Venant-Kirchhoff (VK) material model [28]. The VK material is the simplest hyper-elastic material and holds nonlinear property between the strain and the displacement gradient, and in the meantime obeys Hooke’s law in constitutive relationships.

The deformation of the image pair for registration is assumed to be resulted from a large displacement such as the flexible plates or shell deformed with external forces. The strain relative to the initial (base) configure which is usually called the Green-St.Venant Strain and is defined as

$$\underline{e}=\frac{1}{2}(J_{CG}^{r}-I)$$ (5)

where

$$J_{CG}^{r}=J_{X}^{T}J_{X}={\left[I+\nabla u\right]}^{T}\cdot [I+\nabla u]$$ (6)

where $J_{CG}^{r}$ is the right Cauchy-Green strain tensor, **I** is an identity tensor and **J**_*X*_ is the deformation gradient. Physically, the right Cauchy-Green strain tensor leads to the square of local change in distances due to deformation. By substituting $J_{CG}^{r}$ in Eq (6) to Eq (5), we have

$$\underline{e}=\frac{1}{2}(\nabla u+\nabla u^{T}+\nabla u^{T}\nabla u)=\left[\begin{array}{cc} e_{XX} & e_{XY}\\ e_{YX} & e_{YY} \end{array}\right]$$ (7)

and with the assumption that |∇**u**|<<1, the quadratic term ∇**u** ^*T*^∇**u** can be neglected, which is encountered in the linear elastic finite element model analysis.

The components of **e** can be rearranged as a 3-component strain vector **ε** for the finite element analysis of our elastic model as follows:

$$\varepsilon =\left[\begin{array}{c} e_{1}\\ e_{2}\\ e_{3} \end{array}\right]=\left[\begin{array}{c} e_{XX}\\ e_{YY}\\ 2e_{XY} \end{array}\right]$$ (8)

where

$$e_{m}=h_{m}^{T}g+\frac{1}{2}g^{T}H_{m}g,\;m=1,2,3$$ (9)

where **h**_***m***_ is a 4×1 vector, **H**_*m*_ is a 4×4 sparse symmetric matrix, and **g** is the displacement gradient vector, i.e.,

$$h_{1}=\left[\begin{array}{c} 1\\ 0\\ 0\\ 0 \end{array}\right],h_{2}=\left[\begin{array}{c} 0\\ 0\\ 0\\ 1 \end{array}\right],h_{3}=\left[\begin{array}{c} 0\\ 1\\ 1\\ 0 \end{array}\right]$$ (10)

$$H_{1}=\left[\begin{array}{cccc} 1 & 0 & 0 & 0\\ 0 & 1 & 0 & 0\\ 0 & 0 & 0 & 0\\ 0 & 0 & 0 & 0 \end{array}\right],H_{2}=\left[\begin{array}{cccc} 0 & 0 & 0 & 0\\ 0 & 0 & 0 & 0\\ 0 & 0 & 1 & 0\\ 0 & 0 & 0 & 1 \end{array}\right],H_{3}=\left[\begin{array}{cccc} 0 & 0 & 1 & 0\\ 0 & 0 & 0 & 1\\ 0 & 0 & 0 & 0\\ 0 & 0 & 0 & 0 \end{array}\right]$$ (11)

$$g=\left[\begin{array}{c} \frac{{\partial u}_{X}}{\partial X}\\ \frac{{\partial u}_{Y}}{\partial X}\\ \frac{{\partial u}_{X}}{\partial Y}\\ \frac{{\partial u}_{Y}}{\partial Y} \end{array}\right]=\left[\begin{array}{cc} \frac{\partial }{\partial X} & 0\\ 0 & \frac{\partial }{\partial X}\\ \frac{\partial }{\partial Y} & 0\\ 0 & \frac{\partial }{\partial Y} \end{array}\right]\left[\begin{array}{c} u_{X}\\ u_{Y} \end{array}\right]=\mathrm{LU}$$ (12)

where **u**_*x*_ and **u**_*y*_ are the displacement in *x* and *y* directions.

Assume an elastic continuum without initial stresses of strains, the elastic deformation energy *W*_*e*_ of an elastic body can be expressed as:

$$W_{e}=\frac{1}{2}\int_{\Omega }\sigma^{T}\varepsilon dx$$ (13)

where Ω is the image domain, **σ** is the stress tensor which relates to the Green-St.Venant Strain in the following manner:

$$\sigma =\left[\begin{array}{c} \sigma_{1}\\ \sigma_{2}\\ \sigma_{3} \end{array}\right]=\left[\begin{array}{ccc} D_{11} & D_{12} & D_{13}\\ D_{21} & D_{22} & D_{23}\\ D_{31} & D_{32} & D_{33} \end{array}\right]\left[\begin{array}{c} e_{1}\\ e_{2}\\ e_{3} \end{array}\right]$$ (14)

Eq (14) can be rewritten in a more compact form as **σ = Dε**. For the material with isotropic characteristics, matrix^**D**^ **D** can be determined with the Young’s modulus *E* and Poisson’s ratio *v* as follows:

$$D=\frac{E(1-v)}{\left(1+v)(1-2v\right)}\left[\begin{array}{ccc} 1 & \frac{v}{1-v} & 0\\ \frac{v}{1-v} & 1 & 0\\ 0 & 0 & \frac{1-2v}{2(1-v)} \end{array}\right]$$ (15)

By substituting Eq (8) and Eq (14) to Eq (13), the elastic energy can be rewritten as:

$$W_{e}=\frac{1}{2}\int_{\Omega }\sum_{m,n=1}^{3}W_{mn}dx$$ (16)

where *W*_*mn*_ is usually called the strain energy density and can be expressed as follows:

$$\begin{array}{l} W_{mn}=u^{T}L^{T}h_{m}D_{mn}h_{n}{}^{T}\mathrm{Lu}+\frac{1}{2}u^{T}L^{T}h_{m}D_{mn}u^{T}L^{T}H_{n}\mathrm{Lu}\\ +\frac{1}{2}u^{T}L^{T}H_{m}\mathrm{Lu}D_{mn}h_{n}{}^{T}\mathrm{Lu}+\frac{1}{4}u^{T}L^{T}H_{m}\mathrm{Lu}D_{mn}u^{T}L^{T}H_{n}\mathrm{Lu} \end{array}$$ (17)

where **L** is a differential operator given in Eq (12) and *D*_*mn*_ is the element of matrix **D**.

### 2.3 Elastic Force

According to the Delaunay criteria [29], uniform meshes that are composed of triangular elements Ω^*el*^ are generated for both the reference image *I*_*R*_ and the floating image *I*_*T*_. If the displacement vectors **u**_*i*_^*el*^(*i* = 1,2,3) of the triangular nodes are obtained, the displacement vectors **u**(**x**) of other points in the triangular element can be obtained approximately by the following interpolations:

$$u(x)=\sum_{i=1}^{3}N_{i}^{el}(x)u_{i}^{el}$$ (18)

where *N*_*i*_^*el*^(**x**)(*i* = 1,2,3) is the shape function of an element. Using linear interpolation, the shape functions are defined as the so-called natural coordinates *L*_i_ of the element. i.e.,

$$N_{i}^{el}(x)=L_{i}=\frac{1}{2A^{el}}(a_{i}^{el}+b_{i}^{el}x+c_{i}^{el}y)\;i=1,2,3$$ (19)

With natural coordinates, the area *A* ^*el*^ and the coefficients *a*_*i*_^*el*^, *b*_*i*_^*el*^, *c*_*i*_^*el*^ are all defined as following:

$$A^{el}=\frac{1}{2}\left|\begin{array}{ccc} 1 & x_{1} & y_{1}\\ 1 & x_{2} & y_{2}\\ 1 & x_{3} & y_{3} \end{array}\right|,\;a_{1}=x_{2}y_{3}-x_{3}y_{2},\;b_{1}=y_{2}-y_{3},\;c_{1}=x_{3}-x_{2}$$ (20)

where (*x*_*i*_, *y*_*i*_) *i* = 1,2,3 is the global coordinates of element nodes, and the other coefficients are found by cyclic interchange of the indexes.

Given the expression of the elastic energy, the elastic force ${\hat{F}}_{i}^{el}$ acting on node *i* of element *el* can be derived as:

$${\hat{F}}_{i}^{el}=\frac{1}{2}\int_{\Omega }\sum_{m}\sum_{n}\frac{\partial W_{mn}}{\partial u_{i}^{el}}dx$$ (21)

and by substituting Eq (18) to Eq (17), $\frac{\partial W_{mn}}{\partial u_{i}^{el}}$ can be written as:

$$\begin{array}{l} \frac{\partial W_{mn}}{\partial u_{i}^{el}}=\sum_{j}(B_{i}^{T}h_{m}D_{mn}h_{n}^{T}B_{j}+{\left(B_{i}^{T}h_{m}D_{mn}h_{n}^{T}B_{j}\right)}^{T})u_{j}\\ +\frac{1}{2}\sum_{j}\sum_{k}(B_{i}^{T}h_{m}D_{mn}u_{j}{}^{T}B_{j}^{T}H_{n}B_{k}u_{k}+B_{i}^{T}(H_{n}^{T}+H_{n})B_{j}u_{j}D_{mn}h_{m}^{T}B_{k}u_{k})\\ +\frac{1}{2}\sum_{j}\sum_{k}(B_{i}^{T}(H_{m}+H_{m}^{T})B_{j}u_{j}D_{mn}h_{n}^{T}B_{k}u_{k}+B_{i}^{T}h_{n}D_{mn}u_{j}{}^{T}B_{j}^{T}H_{m}B_{k}u_{k})\\ +\frac{1}{4}\sum_{j}\sum_{k}\sum_{l}(B_{i}^{T}(H_{m}+H_{m}^{T})B_{j}u_{j}D_{mn}u_{k}^{T}B_{k}^{T}H_{n}B_{l}u_{l}+\\ B_{i}^{T}(H_{n}+H_{n}^{T})B_{j}u_{j}D_{mn}u_{k}^{T}B_{k}^{T}H_{m}B_{l}u_{l}) \end{array}$$ (22)

where $B_{i}^{el}=LN_{i}^{el}$, and **L** is a differential operator given in Eq (12). ${\hat{F}}^{el}$as determined in Eq (21) can be assembled to the global $\hat{F}$ in the image domain Ω**.**

### 2.4 External Force

In the framework of FEM, the nodal external force matrix $\hat{R}$ can be assembled by the external force ${\hat{R}}^{el}$ of all element *el*, which is defined as follow:

$${\hat{R}}^{el}=\int_{\Omega }R(x)N^{el}dx$$ (23)

where **R**(**x**) is the external force field subjected to the gradient flows of the matching metric, **N** ^*el*^ is the shape function vector assembled by *N*_*i*_^*el*^(**x**)(*i* = 1,2,3).

Our aim of registration is to find a deformation field so that the registration similarity, whch is represented as spatially encoded mutual information (SEMI) [30], can be maximized. Mathematically, the matching metric increases faster along its gradient direction. Therefore, a plausible choice of the external force would be the gradient flows of the matching metric which would then drive the tissue to deform in a trend increases the matching similarity.

The gradient flows of the similarity metric between reference image r(**x**) and floating image m(**x**) is defined as follow:

$$R(x\prime )=-\frac{1}{N_{s}}\int_{R}\int_{R}\int_{\Omega }L_{s}(r\prime ,m\prime )G_{s}(x,x\prime )\frac{\partial }{\partial u}\phi (r(x)-r\prime ,m(x+u)-m\prime )dxdr\prime dm\prime$$ (24)

where R is the intensity value domain and *G*_*s*_(**x**, **x**′) is the weighting function for spatial information encoding, i.e.,

$$G_{s}(x,x\prime )=\frac{1}{2\pi \gamma^{2}}\text{exp}(-\frac{\left|x-x\prime \right|}{2\gamma^{2}})$$ (25)

where *γ* is the standard deviation, *N*_*s*_ = ∫_Ω_ *G*_*s*_(**x**, **x**′)*d* **x** is the normalization factor and *ϕ*(⋅,⋅) is the Parzen intensity kernel, i.e.,

$$\phi (y_{1},y_{2})=\frac{1}{2\pi \beta^{2}}\text{exp}(-\frac{y_{1}^{2}}{2\beta^{2}})\cdot \text{exp}(-\frac{y_{2}^{2}}{2\beta^{2}})$$ (26)

$$L_{s}(r\prime ,m\prime )=1+\text{log}\frac{p_{s}(r\prime ,m\prime )}{p_{s}(r\prime )p_{s}(m\prime )}$$ (27)

where

$$p_{s}(r\prime ,m\prime )=\frac{1}{N_{s}}\int_{\Omega }G(x,x\prime )\phi (r(x)-r\prime ,m(x+u)-m\prime )dx$$ (28)

where *β* is the smoothing parameter for the Parzen estimates, *p*_*s*_(*r* ^’^) and *p*_*s*_(*m* ^’^) are the region marginal intensity probability density of the reference image and floating image respectively.

### 2.5 Hierarchical Registration Strategy

**Fig 2:**
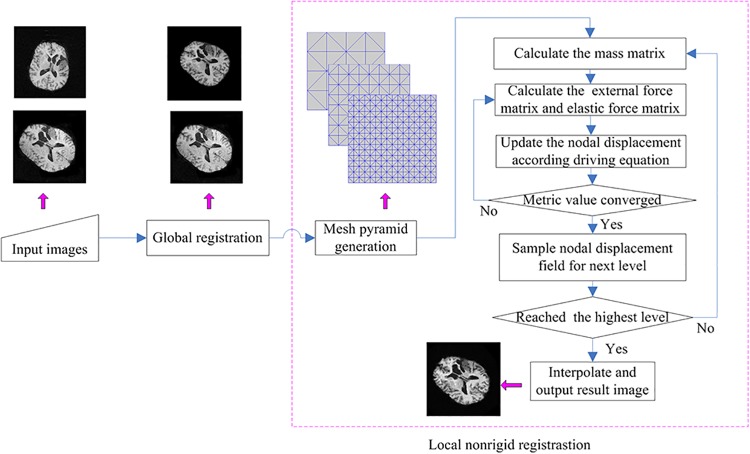
The framework of our image registration algorithm.

For a more efficient implementation of our proposed registration method for images with large deformation, a hierarchical strategy (often referred to as the “global-to-local” strategy) was adopted in our registration procedure. A schematic illustration of the block diagram of our hierarchical registration method is shown in Fig 2.

The global registration is to find the transformation matrix maximizing the similarity metric that is mutual information in our paper [31]. After the global registration, a subsequent local non-rigid registration was performed based on our proposed NFEM. We constructed the coarse-to-fine registration pyramid by firstly performing the filtering of the images with a Gaussian low pass filter for noise reduction and then setting uniform mesh nodes using the predefined element size to generate the mesh pyramid.

### 2.6 Parameters Setting

We set three types of parameters in our method. The first one is the elastic material related parameters. For simplicity, we considered our model as a homogeneous isotropic hyperelastic model and we fixed the elastic modulus *E* = 100kPa and the Poisson’s ratio *v* = 0.45 as in [32]. The second type is the parameters related to the optimization procedure including the number of hierarchical levels, the element sizes and the time step for the explicit integration scheme. In our experiments, the spatial resolution of the images is 256×256. Upon pyramid generation, excessively larger number of nodes would increase computational requirement while excessively smaller number of nodes would lead to unacceptable loss of information. In our experiments, meshes with 64×64, and 32×32 nodes were used to construct a two–level multi-resolution pyramid for better balance of computational efficiency and registration accuracy.

Since the explicit integration scheme for the nonlinear elastic model is only conditionally stable, a thorough analysis about it has been presented by determining a suitable time step. Similar to the procedure as in [33], we chose △*t* = 0.004, the value of the damping coefficient and the mass density were deduced according to the predefined spectral radius *ρ* ^*^ (*ρ* ^*^ = 0.99) [33]. These parameters are summarized in Table 1.

**Table 1:** Parameters for our proposed method.

The elastic modulus *E*	Poisson’s ratio *v*	The mass density *ρ*	Damping coefficient *α*	Integration time step Δ*t*
100kPa	0.45	1000 *kg*/*m* ^3^	5	0.004

The third type of parameters is for SEMI metric including the size of the local region denoted as the diameter *d*, the standard deviation *γ* and the smoothing parameter *β* for the Parzen estimates. As analyzed in [30], there was a tradeoff between the global robustness and the local accuracy. The large local region contributes more information for estimating the joint intensity probability density, while the small local region contributes the better accuracy in the local region. For obtaining optimized values for the region diameter *d*, analysis of registration accuracy over 20 abdominal CT image pairs for different *d* values was conducted.

### 2. 7 Materials

Evaluations of our nonlinear finite element model (NFEM) were performed on 30 clinical CT image pairs with artificial deformations. These abdominal and chest CT images were acquired from the First Affiliated Hospital of Soochow University and used as the reference images. 10 B-Spline based small synthetic deformation fields and 10 large synthetic deformation fields were used to warp the reference image to generate floating images for the nonrigid registration. In order to compare our method as well as the LFEM and the DFEM methods with the RBM method, 10 different non-uniform artificial fields were used to generate ten floating images, instead of the B-Spline based predefined fields used above. The deformation exceeding 15 pixels in local regions was considered as “large” deformation and was included to validate our proposed NFEM algorithm.

We also evaluated the performance of our proposed algorithm for clinical images including 10 chest CT image pairs and 20 brain MRI image pairs. The chest CT image pairs were acquired from two different treatment periods of 10 patients in the First Affiliated Hospital of Soochow University. The brain MRI images from different healthy volunteers were collected and made available by the CASILab at The University of North Carolina at Chapel Hill and distributed by the MIDAS Data Server at Kitware, Inc [34]. We randomly selected 21 T1-Flash Images and chose one T1-Flash image as the reference and the rest 20 T1-Flash images as floating images.

### 2.8 Ethics Statement

The study about CT images was carried out according to the Helsinki Declaration and approved by the ethical committee of the First Affiliated Hospital of Soochow University. The need for informed consent was waived, because the data used in this study had already been collected for clinical purposes. Furthermore, the present study did not interfere with the treatment of patients and the database was organized in a way that makes the identification of an individual patient impossible. The study about MR images was carried out according to the Helsinki Declaration and approved by the ethical committee of the University of North Carolina at Chapel Hill. All subjects provided signed consent allowing images to be made publicly available on the website. The database was organized in a way that makes the identification of an individual patient impossible.

### 2.9 Registration Accuracy Assessment

To evaluate the proposed NFEM algorithm for non-rigid registration, we compared it with 1) the conventional LFEM registration algorithm; 2) DFEM registration algorithm; and 3) the robust block matching (RBM) based registration software which was called “Nifty_Reg” and developed at University College London containing programs to perform rigid, affine and the RBM algorithms for nonlinear registration [35].

Evaluation was carried out by visual assessment in subtraction images between the reference image and the floating image after registration and also by three popular measures: the mean square difference (MSD), the normalized correlation (NC) and the normalized mutual information (NMI). Furthermore, for the registration of the images with artificial deformations, 40 random foreground points were automatically selected from the reference images. Then the mean and max distance of the corresponding points in the image pairs before and after registration was calculated to measure the registration accuracy.

## Results

**Fig 3:**
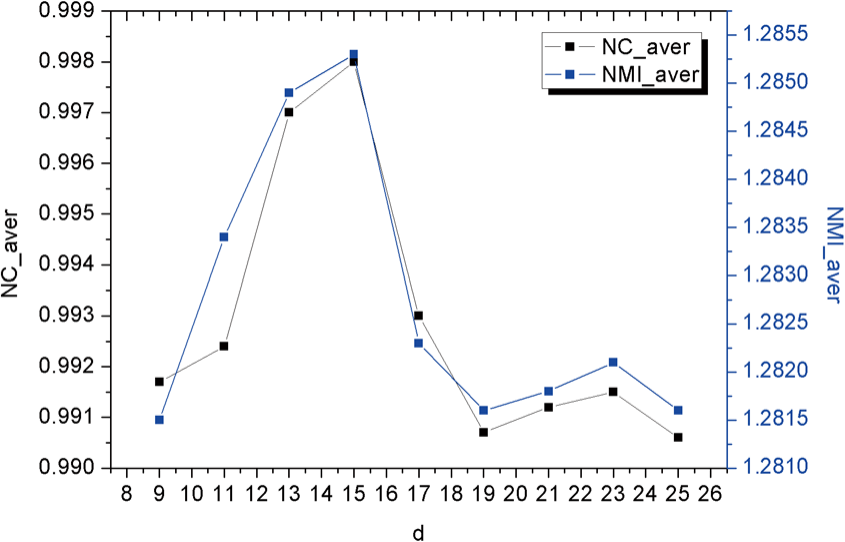
The average normalized correlation (NC_aver) and normalized mutual information (NMI_aver) with different d values. (a) Values at level 0 with the mesh resolution 8×8; (b) Values at level 1 with the mesh resolution 4×4.

Curves in Fig 3 show the impact of parameter *d* on the average values of NC and NMI. We found *d* = 15 contributed the highest NC and NMI values. The standard deviation *γ* was set to a small value, typically *γ* = 0.25*d.

### 3.1 Registration Results of Medical Images with Uniform Deformations

**Fig 4:**
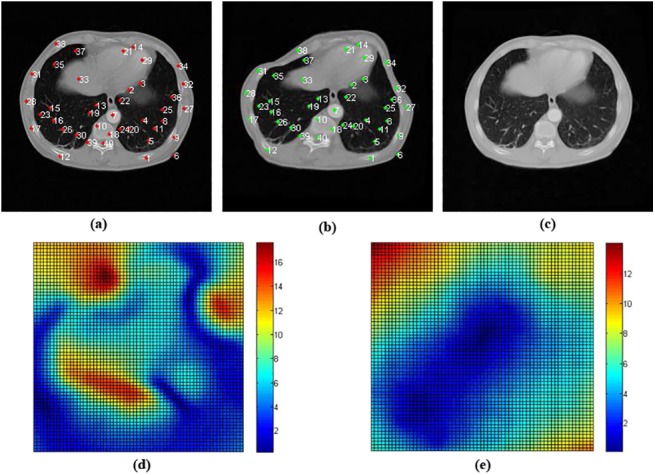
The reference image, corresponding floating image and the registration results. (a) The reference image attached 40 points (in red color and with white indexes) as registration ground truth; (b) and (c) are the floating image attached 40 points (in green color and with white indexes); (d) and (e) are the color images of deformations.

Fig 4 illustrates two examples of the image pairs for registration, where Fig 4B is the floating image derived from reference image Fig 4A upon large uniform artificial deformation, and Fig 4C is the floating image derived from Fig 4A upon relatively small uniform deformation. 40 random positions in the object region chosen as label points were marked in red in Fig 4A. The corresponding positions in the floating image were acquired from pre-defined artificial large deformation fields and marked in green in Fig 4B; As shown in Fig 4C, the amount of this kind of deformation was set to exceed 15 pixels in local regions. Fig 4D and 4E show the color images of the distance of a point in the reference to its counterpart in the floating image to display the deformation degree in local regions.

**Fig 5:**
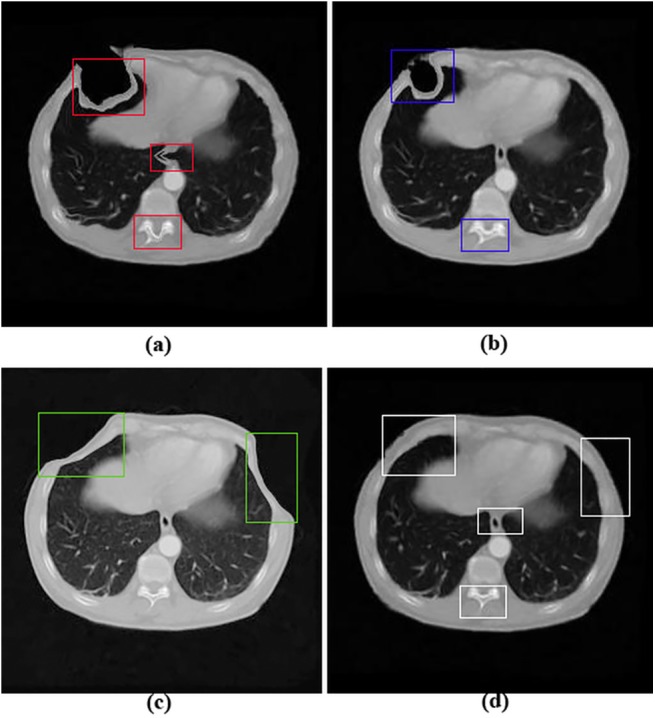
Registration results of different methods upon artificial image pairs with uniform large deformations. (a) the registration result corresponding to the floating image with the LFEM method; (b) the registration result with DFEM method; (c) the registration result with RBM method; (d) the registration result with NFEM method. Squares in images highlight the local differences.

The visual assessment and comparison of the registration results by observing the sub-regions in the squares were performed as shown in Fig 5. The more corresponding and similar the registration results and the reference images are, the more capable the algorithm of tackling large deformations. As demonstrated in Fig 5D, our registration result was more corresponding to the reference image.

Table 2 shows the quantitative evaluations of different methods on the ten pairs of images with large uniform deformations in terms of the mean distance (mean_x and mean_y) and maximal distance (max_x and max_y) between true and recovered deformation in the x and y direction. The mean values of the mean and max distances of our method were the lowest when compared to the mean values from other three methods, which indicated that our method achieved the highest registration accuracy. Student’s t-test (p-values <0.05) validated this improvement was statistically significant.

**Table 2:** Mean distance and maximal distance of different algorithms for images with large deformations (Mean value (95%CI)).

Distances	RBM	LFEM	DFEM	NFEM	*p*- value<0.05 (NFEM vs RBM, LFEM and DFEM respectively)
***x***_***mean***_	0.476 (0.318,0.635)	0.833 (0.470,1.197)	0.733 (0.383,1.083)	0.320 (0.137,0.503)	0.04,2.34E-9,7.37E-4
***y***_***mean***_	0.4430 (0.2326,0.6533)	0.961 (0.577,1.344)	0.950 (0.618,1.282)	0.326 (0.215,0.437)	0.02,4.16E-6,4.61E-6
***x***_***max***_	5.735 (3.788,7.682)	6.037 (4.566,7.508)	5.698 (4.106,7.290)	3.110 (1.093,5.127)	0.009,0.006,0.01
**y**_**max**_	4.971 (3.600,6.342)	6.734 (4.938,8.530)	5.735 (4.579,6.891)	4.078 (2.153,6.003)	0.01,2.49E-4,0.002

CI represents confidence interval.

The comparisons of our proposed NFEM method with other three methods in terms of MSD, NC and NMI (shown in Table 3) demonstrated that the mean values of NC and NMI using our NFEM was greater than the corresponding values using other three methods, and consistently, the mean value of MSD using NFEM was the lowest. Student’s t-test (p-values <0.05) consistently validated our method statistically outperformed its counterpart comparison methods.

**Table 3:** The average metric values of different algorithms (Mean value (95%CI)).

Metrics	RBM	LFEM	DFEM	NFEM	*p*–value<0.05 (NFEM vs RBM, LFEM and DFEM respectively)
**MSD**	411.4 (108.77,714.05)	164.26 (109.55,218.97)	137.21 (32.06,242.36)	41.96 (28.22,55.69)	4.14E-10, 3.06E-7,1.34E-6
**NC**	0.9532 (0.9105,0.9959)	0.9875 (0.9792,0.9959)	0.9830 (0.9671,0.9989)	0.9958 (0.9939,0.9977)	1.34E-9,7.87E-4,7.87E-4
**NMI**	1.2701 (1.2348,1.3054)	1.2397 (1.2241,1.2553)	1.2424 (1.2135,1.2713)	1.2962 (1.2848,1.3076)	6.95E-4,9.77E-6,1.28E-5

CI represents confidence interval.

**Fig 6:**
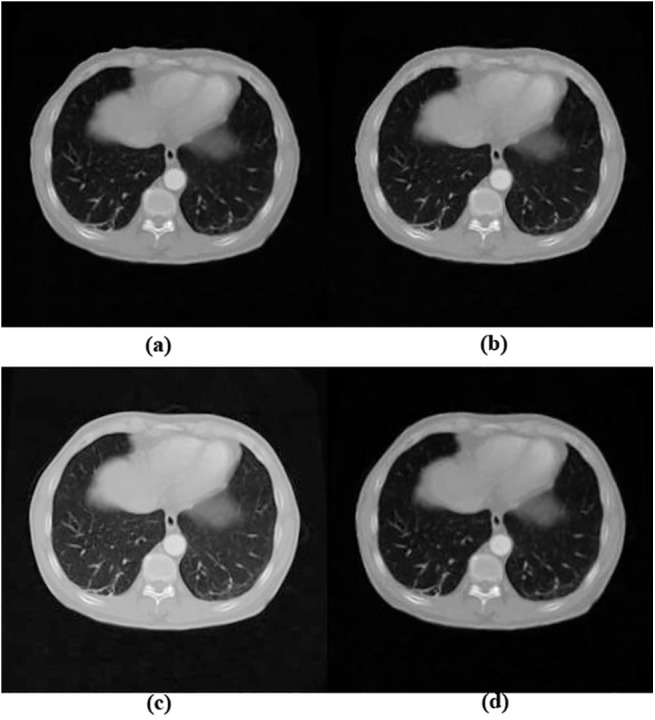
Registration results of different methods upon artificial image pairs with uniform small deformations. (a) the registration result of the LFEM method; (b) the registration result of DFEM method; (c) the registration result of RBM method; (d) the registration result of NFEM method. Squares in imags highlight the local differences.

The quantitative validation on ten pairs of images with small uniform deformations is displayed in Table 4. Table 4 summarized the mean distances and different metrics for 10 image pairs with small deformation and demonstrated that the RBM achieved the best registration results followed by our NFEM. In comparison, although there were no obvious matching errors in the registration results shown in Fig 6, LFEM and DFEM algorithms slightly fell behind regarding the registration accuracy because of the infinitesimal deformation assumption.

**Table 4:** Mean distance, maximal distance and metrics of different algorithms.

Distance and Metrics	RBM	LFEM	DFEM	NFEM
***x***_***mean***_	0.1812	0.6317	0.3503	0.3444
***y***_***mean***_	0.2030	0.7219	0.4169	0.3615
***x***_***max***_	1.6530	2.7389	2.3483	2.2977
**y**_**max**_	3.0781	3.1450	2.849	2.7443
**MSD**	85.07	91.28	86.06	80.36
**NC**	0.9957	0.9883	0.9899	0.9904
**NMI**	1.3332	1.2520	1.2736	1.2839

### 3.2 Registration Results of Medical Images with Non-uniform Deformations

**Fig 7:**
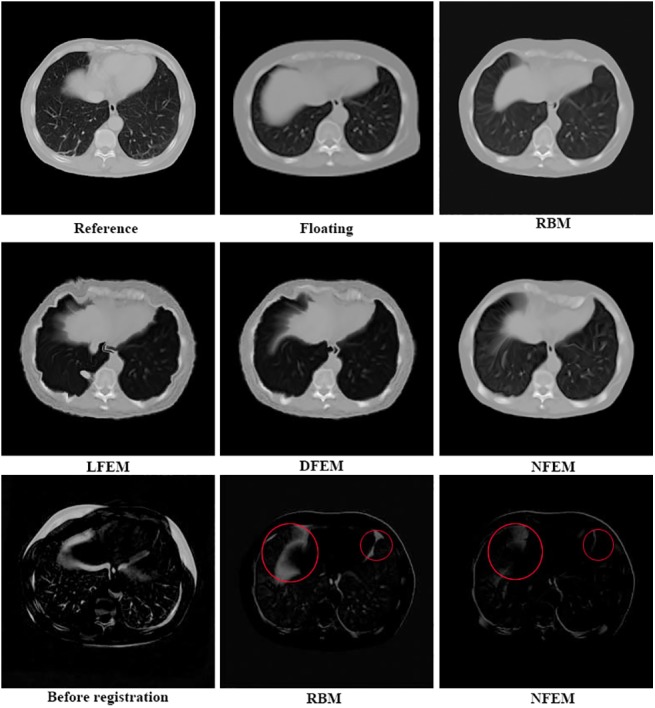
Reference image, the corresponding floating image and registration result. The first two rows present the reference image, the floating image and the registration results with the RBM method, LFEM method, DFEM method and NFEM method. The bottom row shows the subtraction images before registration and the ones using RBM and NFEM methods. Circles in (e) and (f) highlight the local differences in the subtraction image.

Fig 7 illustrates the examples of the registration results over the image pairs with non-uniform artificial fields. Circles in the subtraction images highlight the difference in the local regions in the results from different methods. The performance comparisons among different methods in term of MSD, NC and NMI are illustrated in Table 5. In addition, the Student’s t-test (p-values <0.05) shown in Table 5 indicated that our NFEM method significantly outperformed other three methods. The visual assessment displayed as the subtraction images in Fig 7 further consolidated the same conclusion.

**Table 5:** The average metric values of different algorithms (Mean value (95%CI)).

Metrics	RBM	LFEM	DFEM	NFEM	*p*- value<0.05 (NFEM vs RBM, LFEM and DFEM respectively)
**MSD**	388.99 (280.12,497.86)	544.89 (409.02,680.75)	472.09 (390.39,553.80)	165.75 (130.53,200.98)	8.36E-8,8.13E-10,5.26E-9
**NC**	0.9434 (0.9258,0.9610)	0.9416 (0.9291,0.9541)	0.9506 (0.9434,0.9578)	0.9802 (0.9774,0.9830)	1.41E-10,9.19E-11,9.79E-10
**NMI**	1.2616 (1.2548,1.2685)	1.2372 (1.2308,1.2437)	1.2384 (1.2334,1.2434)	1.2811 (1.2762,1.2861)	4.79E-6,4.62E-9,5.81E-9

### 3.3 Registration Results of Real Medical Images

**Fig 8:**
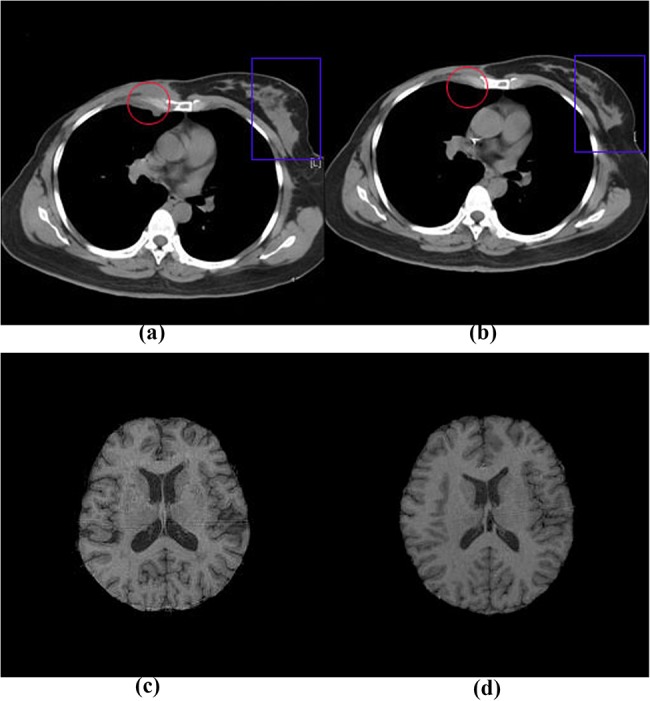
Medical images examples for method evaluation. (a) reference image of chest CT image pairs,(b) floating image of chest CT image pairs, (c) reference image of brain MR image pairs, (d) floating image of brain MR image pairs.

Fig 8 illustrates two example image pairs used in our registration method evaluation. Fig 8A is a pre-chemotherapy image and Fig 8B is the post-chemotherapy image after about three month’s treatment. Red circles in Fig 8A and 8B highlight the local variations because of the chemotherapy, while the variation in blue squares is resulted from the deformation of chest tissues in the different body position. Fig 8C and 8D are a pair of T1-Flash images from different subjects.

**Fig 9:**
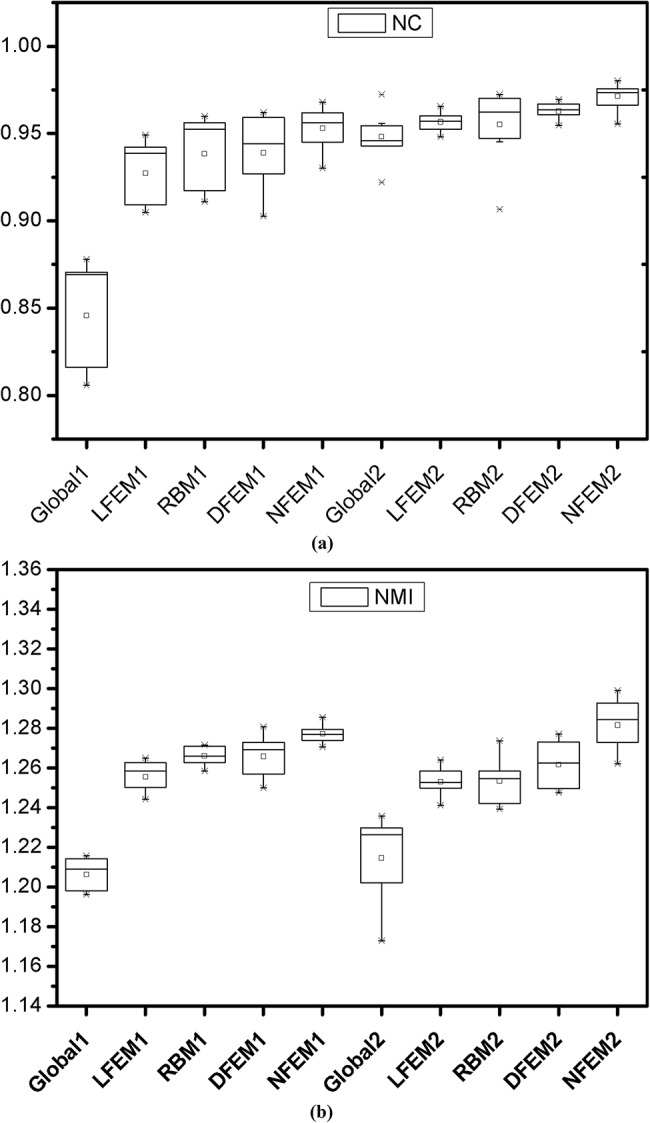
Statistic box-plots of registration results in terms of NC and NMI. (a) NC parameters for box-plots. (b) NMI parameters for box-plots. In CT image registration cases, these methods are represented by appending a suffix 1 (e.g. Global1) to the name abbreviations, while in MRI image cases, the suffix 2 is added to their name abbreviations (e.g. Global2).

The statistical performance in terms of NC and NMI for different methods in both chest CT and brain MRI cases is shown in Fig 9 where these two matching metrics are illustrated by means of statistic box-plots. The t-tests were implemented and results are illustrated in Table 6 and Table 7, which indicated that, at the 0.05 level, the mean value for NC and NMI of all registration validation using method NFEM is significantly greater than the ones using Global, LFEM, DFEM, RBM method.

**Table 6:** The average metric values of different algorithms for CT chest images (Mean value (95%CI)).

Metrics	NC	NMI
**Global**	0.8457(0.8210,0.8704)	1.2062(1.1997,1.2126)
**RBM**	0.9384(0.9218,0.9550)	1.2661(1.2623,1.2698)
**LFEM**	0.9272(0.9130,0.9413)	1.2555(1.2493,1.2617)
**DFEM**	0.9389(0.9212,0.9567)	1.2635(1.2534,1.2736)
**NFEM**	0.9530(0.9429,0.9632)	1.2713(1.2631,1.2795)
***p*-value (NFEM vs RBM, LFEM and DFEM respectively)**	<0.05	<0.05

**Table 7:** The average metric values of different algorithms for MRI brain images(Mean value (95%CI)).

Metrics	NC	NMI
**Global**	0.9482(0.9379,0.9586)	1.2146(1.1986,1.2306)
**RBM**	0.9553(0.9392,0.9713)	1.2534(1.2445,1.2623)
**LFEM**	0.9567(0.9525,0.9608)	1.2530(1.2472,1.2588)
**DFEM**	0.9628(0.9587,0.9668)	1.2617(1.2523,1.2712)
**NFEM**	0.9715(0.9656,0.9773)	1.2815(1.2719,1.2912)
***p*- value (NFEM vs RBM, LFEM and DFEM respectively)**	<0.05	<0.05

All evaluations were implemented with C on Windows 7 operating system and performed on a DELL desktop with Intel(R) Core(TM) i7-4770 @ 3.4GHz CPU. Using the aforementioned two-level pyramid, the total freedom degree for computing the displacement field was 10240. The average computation time using LFEM was 3 minutes and 35 seconds and the one using DFEM was 4 minutes and 3 seconds. The average computation time using our NFEM method was 5 minutes and 26 seconds.

## Discussion

Complicated nonlinear characteristics of the soft tissues or the tremendous deformations introduced by the internal organ movements or treatment interventions pose significant challenges for the current non-rigid image registration algorithms. Underlying linear hypotheses of these algorithms are one of the foremost reasons to underpin the requirement for further improvement of these nonlinear methods. We took into account the geometric nonlinearity of elastic body model. In our algorithm, the elastic force was modeled as the derivative of the deformation energy with respect to the nodal displacement vectors of the finite element; the external force was derived from the registration similarity gradient flow.

We validated our algorithm firstly on image pairs with artificial deformations and then on real CT and MRI images. We compared our NFEM method with three other methods: 1) the conventional LFEM registration algorithm; 2) DFEM registration algorithm; and 3) the robust block matching (RBM) algorithm.

To validate the performance of our algorithm when registering images with large deformations, we conducted experiments on ten pairs of images with large artificial deformations. The visual assessment and comparison of the registration results were performed by observing the sub-regions in the squares as shown in Fig 5. As demonstrated in Fig 5D, our registration result was more corresponding to the reference image, which indicated that our proposed NFEM algorithm outperformed other three algorithms. The better registration results from our algorithm were mainly due to the contribution of the internal elastic force that took into account the geometrical nonlinearity to compensate for the local large displacements.

The quantitative evaluations of different methods (as shown in Table 2 and Table 3) on the ten pairs of images with large uniform deformations consistently demonstrated that our method statistically outperformed its counterpart comparison methods. These better statistical results from our algorithm also indicated that the nonlinear internal elastic force played the positive role in the registration.

The quantitative validation on ten pairs of images with small uniform deformations as displayed in Table 4 demonstrated that the RBM achieved the best registration results followed by our NFEM. The better accuracy from RBM method benefited from the fact that RBM was a B-Spline based method and it was therefore inherently more suitable for handling the small uniform deformations generated by B-Spline interpolation. In comparison, although there were no obvious matching errors in the registration results shown in Fig 6, LFEM and DFEM algorithms slightly fell behind regarding the registration accuracy because of the infinitesimal deformation assumption. The registration results in Table 2 and Table 4 demonstrated that our method was more robust for the cases with both large deformations and small deformations.

Meanwhile, 10 corresponding floating images were generated by pre-defined non-uniform artificial fields to investigate the feasibility of our method for estimating large non-uniform deformations. The performance of different methods was compared in terms of MSD, NC and NMI. The Student’s t-test (p-values <0.05) shown in Table 5 indicated that our NFEM method significantly outperformed other three methods. The visual assessment displayed as the subtraction images in Fig 7 further consolidated the same conclusion. The nonlinear elasticity modeling via Green-St.Venant strain measure contributed to the improved deformation estimation and hence led to the improved registration accuracy for the images with non-uniform deformations.

In addition to the evaluations of our proposed algorithm on artificially deformed images, we validated our algorithm on clinical CT and MRI images as shown in Fig 8. The statistic box-plots illustrated in Fig 9 demonstrated that our method was able to effectively compensate the deformations. Statistical analysis as illustrated in Table 6 and Table 7 indicated that, at the 0.05 level, the mean value for NC and NMI of all ten registration validation using method NFEM is significantly greater than the ones using Global, LFEM, DFEM, RBM method, which demonstrated that our proposed NFEM algorithm outperformed other methods for registering real clinical image pairs and would be potentially usable for assessing disease progression or patient response to treatment.

While our method achieved improved registration accuracy compared to other linear elastic registration models, it has a tradeoff in regard to the computation time. Theoretically, explicit integration in our NFEM method should contribute to improve the computational efficiency. However, updating the relatively complicated elastic force in the iterations was computationally costly. The convergence towards the steady-state solution would be increased if the integration time step could be adaptively updated during iterations.

## Conclusion

In this paper, a novel physics-based nonlinear registration algorithm is proposed based on the FEM elastic body model in the framework of hierarchical strategy. The novelty of this algorithm is the inclusion of nonlinearity between the strain and the displacement gradient based on St.Venant-Kirchhoff model. This nonlinear model is represented as a second order differential equation to obtain equilibrium between the internal and the external forces. Experimental validation on images with artificial deformations and real medical images demonstrated that our nonlinear FEM elastic model outperformed the traditional LFEM model, DFEM method, and RBM method in terms of the quantitative and qualitative analysis.
